# Lyophilization Prior to Homogenisation and Extraction Increases Membrane Protein Detection in Gram-Negative Bacterial Proteomic Analyses

**DOI:** 10.3390/proteomes14030035

**Published:** 2026-07-15

**Authors:** Breyer Woodland, Luke A. Farrell, Matthew B. O’Rourke, Matthew P. Padula

**Affiliations:** School of Life Sciences and Proteomics and Metabolomics Core Facility, Faculty of Science, University of Technology Sydney, Ultimo, NSW 2007, Australia; breyerwoodland@outlook.com (B.W.); lukefarrell98@outlook.com (L.A.F.); matthew.orourke1@gmail.com (M.B.O.)

**Keywords:** antimicrobial resistance, membrane proteins, proteomics, bacterial protein extraction, multi-drug resistance bacteria

## Abstract

Background: Multi-drug resistant Gram-negative bacteria (GNB) are major contributors to the antimicrobial resistance (AMR) burden. AMR mechanisms are primarily mediated by proteoforms; therefore, proteomic analyses of GNB offers a significant advantage in understanding the mechanisms of AMR. A large portion of these mechanisms are mediated by membrane proteins; however, they are often difficult to extract due to their hydrophobic nature and complex interactions with other components of the cell membrane. To extract the greatest number of proteoforms, an efficient homogenisation protocol is required to effectively disrupt the rigid cell wall and membrane. Methods: Using *Escherichia coli*, *Klebsiella pneumoniae*, *Acinetobacter baumannii* and *Pseudomonas aeruginosa*, we systematically compared the extraction efficiency of bead-beating with flash frozen and lyophilized cell pellets. Results: We demonstrate that lyophilization improves bead-beating extraction methods by increasing the detection of membrane proteins. We detected numerous unique membrane proteins in each bacterial isolate, including ABC transporters and proteins involved in lipopolysaccharide synthesis, when lyophilizing prior to bead-beating, compared to only flash-freezing. Conclusions: As membrane proteins play a central role in AMR mechanisms, this improvement in their isolation and identification will aid in understanding the resistance and molecular mechanisms associated with multi-drug resistant GNB.

## 1. Introduction

Gram-negative bacteria (GNB) are responsible for a large number of infections in humans and animals. Their ability to rapidly develop resistance to antimicrobials poses substantial economic and public health threats, emphasizing the need for a comprehensive understanding of resistance and pathogenic mechanisms in order to develop novel therapeutics and diagnostics [1,2]. These mechanisms are primarily mediated by proteoforms, and therefore proteomic analyses of GNB offers a significant advantage in understanding bacterial pathogenesis, the mechanisms of antimicrobial resistance (AMR), and the identification of virulence factors [3,4,5,6].

Comprehensive proteomic analyses are dependent on efficient, robust sample preparation that extracts the greatest number of proteoforms. Numerous aspects of such extraction protocols have been assessed for extracting whole proteomes from Gram-negative bacteria, including: (1) cell lysis and homogenisation techniques, (2) protein extraction and solubilization buffers, and (3) methods to remove unwanted analytes or contaminants (i.e., lipids, nucleic acids, salts, etc.) that may interfere with downstream analyses [7,8,9,10,11,12,13]. While GNB are relatively easy to lyse compared to other microbes due to the thinner layer of peptidoglycan in the cell wall, an effective homogenisation protocol is still required to effectively disrupt the rigid cell wall and cell membrane into small enough pieces to more efficiently extract proteoforms. Broadly, cell lysis and homogenisation techniques include mechanical disruption, such as ultrasonication or bead-beating, and non-mechanical disruption such as freeze-thaw, enzymatic, and detergent-based methods, or a combination of these [14]. Previously, it has been demonstrated that bead-beating results in improved cell lysis and extraction yields compared to alternative mechanical disruption methods, such as ultrasonication or detergent-only based methods [9,11,15]. As initially demonstrated by Rout et al. with yeast, bead-beating results in fragmentation of the cell and cell wall into extremely small fragments, often consisting of a single molecule of a membrane protein that can be more efficiently solubilized [16]. Furthermore, Abele et al. demonstrated that compared to detergent-based methods, such as 2% sodium deoxycholate (SDC), 2% sodium dodecyl sulfate (SDS), or 8M urea, applying an additional mechanical disruption method, such as bead-beating, improved cell lysis efficiency [11]. However, the efficiency of bead-beating is dependent on various factors, including bead size, frequency and speed of oscillations, and cell membrane or cell wall structural characteristics [17,18]. Lyophilizing cell pellets has been suggested to improve mechanical disruption methods, such as bead-beating, due to weakened cellular integrity, therefore making them more susceptible to lysis [19]. Compared to flash frozen cells, Unterlander et al. demonstrated that lyophilizing harvested microalgae cells prior to protein extraction increased the yield of soluble proteins [19]. However, there has been no comparison on the efficiency of protein extractions with lyophilized and flash frozen bacterial cells. Using multi-drug resistant isolates, *Escherichia coli*, *Klebsiella pneumoniae*, *Acinetobacter baumannii* and *Pseudomonas aeruginosa,* we systematically compare the homogenisation and resulting extraction efficiency of flash frozen and lyophilized cell pellets from GNB prior to bead-beating.

Using shotgun proteomics, we demonstrate that lyophilization further improves bead-beating extraction methods by increasing protein extraction yield and increasing the detection of hydrophobic, membrane proteins (i.e., open reading frame (ORF) products), including multi-drug efflux pumps and proteins involved in lipopolysaccharide (LPS) synthesis. Membrane proteins play a central role in AMR mechanisms, therefore improving the capacity to identify them can further aid in understanding the resistance mechanisms and molecular mechanisms associated with multi-drug resistant GNB.

## 2. Materials and Methods

### 2.1. Strains and Reagents

The *E. coli* strain used in this study is strain MG1655, the laboratory reference strain of *E. coli* K-12, hence referred to as Coli K12. The *K. pneumoniae* isolates used in this study are two multi-drug resistant clinical isolates, hence referred to as KC32 and KC89. The *A. baumannii* strain is a multi-drug resistant environmental strain, hence referred to as AB472 [20]. The *P. aeruginosa* strain used in this study is strain PAO1, the laboratory reference strain of *P. aeruginosa*, hence referred to as PAO1.

All other consumables were of analytical or microbiology grade. Tryptone, yeast extract, sodium chloride, urea, thiourea, 3-(4-Heptyl)phenyl-3-hydroxypropyl)dimethylammoniopropanesulfonate (C7BzO), Benzonase^®^ Nuclease, tributylphosphine (TBP), dithiothreitol (DTT), trifluoroacetic acid, acetonitrile, tris hydrochloride (Tris-HCl), and Roche cOmplete™ Mini EDTA-free Protease Inhibitor Cocktail tablets were purchased from Sigma Aldrich. BeadBug^TM^ homogenization tubes (2 mL capacity) and 3 mm zirconium beads from Benchmark Scientific (Sayreville, NJ, USA) were purchased from Sigma Aldrich (St. Louis, MO, USA). Acrylamide was purchased from Bio-Rad (Hercules, CA, USA). Mass Spectrometry Grade Trypsin Gold was purchased from Promega (Madison, WI, USA). Milli-Q water was used throughout.

### 2.2. Culture Conditions

Bacterial isolates were cultured in Lysogeny Broth (LB) at 37 °C and 200 rpm in five biological replicates (with the exception of KC89, where *n* = 3) and grown to late exponential/early stationary phase (OD600 ≈ 1.30). Each culture was divided into two 20 mL aliquots, followed by harvesting by centrifugation at 10,000× *g* for 5 min. The supernatant of each aliquot was discarded. The cell pellet of one aliquot from each biological replicate was lyophilized using a Christ Alpha 2–4 LDplus Laboratory Freeze Dryer (Osterode am Harz, Germany), and the cell pellet of the second aliquot from each biological replicate was flash frozen with liquid nitrogen.

### 2.3. Protein Homogenisation and Extraction

The lyophilized and flash frozen cell pellets were disrupted and pulverized using a Benchmark Scientific BeadBug Benchtop Microtube Homogeniser for 3 × 30 s at 4000 rpm with 5 zirconium beads per tube, and the sample was placed on ice for 30 s between rounds. The resulting pulverized cell pellets were suspended in UTC7 extraction buffer (7 M Urea, 2 M Thiourea, 1% C7BzO, 100 mM Tris-HCl [pH 8.8]) supplemented with 1X Roche cOmplete™ Mini EDTA-free Protease Inhibitor (PI) Cocktail and 50 mM LiCl, then sonicated in a bath sonicator for 10 min [21].

Both extracts were treated with 1 μL benzonase, bath sonicated for a further 10 min, followed by a 50 min incubation period at room temperature to shear DNA, thereby reducing the viscosity of the extracts. The extracts were reduced with 100 mM DTT + 5 mM TBP for 1 h, followed by alkylation with 220 mM of acrylamide for 1 h, at room temperature [22]. Insoluble cellular debris was removed by centrifugation for 10 min at 16,873× *g*.

### 2.4. Quantification, Digestion and Peptide Clean Up

Protein quantification and normalization were performed using a paper-based assay, as previously described [23]. Fifty micrograms of protein was diluted 10-fold with 100 mM Tris-HCl to reduce the concentration of urea to >1 M and digested with 500 ng sequencing grade trypsin (100:1 protein:enzyme ratio) at 37 °C for 16 h. Peptides were recovered using SDB-RPS-based STAGE tips, as previously described [24,25]. Eluted peptides (10 μg) were evaporated to dryness using a Savant^TM^ DNA 120 SpeedVac Concentrator (Thermo Fisher Scientific, Waltham, MA, USA) and reconstituted in 100 µL of MS loading solvent (2% (*v*/*v*) acetonitrile, 0.2% (*v*/*v*) trifluoroacetic acid) for a final concentration of 100 ng/µL.

### 2.5. Liquid Chromatography-Tandem Mass Spectrometry (LC-MS/MS)

Using a Waters Acquity M-class nanoLC system, 500 ng of the sample in 5 µL was loaded at 15 μL/min for 2 min onto a nanoEase Symmetry C18 trapping column (180 μm × 20 mm; Waters, MA, USA) before being washed onto an Aurora Elite TS C18 column (150 mm × 75 μm ID, 1.6 μm C18 particle size; Ion Opticks, Fitzroy, Victoria, Australia) heated to 45 °C. Peptides were eluted from the column and into the source of a Q Exactive Plus mass spectrometer (Thermo Fisher Scientific) using the following gradient: 5–30% MS buffer B (100% acetonitrile) and 95–70% MS buffer A (0.2% formic acid in water) over 120 min, 30–80% MS buffer B over 2 min, 80% MS buffer B for 2 min, and 80–5% over 2 min. The eluting peptides were ionized at 2100 V. A data-dependent MS/MS (dd-MS2) experiment was performed, with a survey scan of 350–1500 *m*/*z* performed at 70,000 resolution for peptides of charge state 2+ or higher with an AGC target of 3e6 and maximum injection time of 50 ms. The top 12 peptides were selected and fragmented in the HCD cell using an isolation window of 1.4 *m*/*z*, an AGC target of 1 × 10^5^ and a maximum injection time of 100 ms. Fragments were scanned in the Orbitrap analyser at 17,500 resolution and the product ion fragment masses measured over a mass range of 120–2000 *m*/*z*. The mass of the precursor peptide was then excluded for 5 s.

### 2.6. Data Analysis

The MS raw files were searched using Peaks Studio 13 (Bioinformatic Solutions Inc., Waterloo, ON, Canada) against their respective UniProt proteome databases (*Escherichia coli* K12 (UP000000625, date accessed 10 October 2025), *Klebsiella pneumoniae* HS11286 (UP000007841, date accessed 11 August 2025), *Acinetobacter baumannii* (UP000498640, date accessed 4 May 2026) and *Pseudomonas aeruginosa* (UP000002438, date accessed 4 May 2026)), and a database of common contaminants with the following parameters: Precursor mass error tolerance: 10.00 ppm. Fragment mass error tolerance: 0.02 Da. Enzyme: Trypsin. Maximum missed cleavages: 4. Digest-mode: Semi-specific. Peptide length range: 6–45. Fixed modifications: none. Variable modifications: Propionamide (Cysteine), Oxidation (Methionine), Deamidation (Asparagine and Glutamine), Carbamylation (N-terminal Lysine). Maximum variable PTM per peptide: 4. Peptide spectrum match (PSM) false discovery rate (FDR): 1.0%. Protein Group FDR: 1.0%. Unique peptides: ≥1. For relative quantification, Peaks Studio label-free quantification was used with the following parameters: Normalization method: total ion chromatogram (TIC). Normalization strategy: retention time (RT) dependent. Top N peptides used for quantification: 3.

Exports from Peaks Studio search results were processed in R Studio (version 2025.09.1) using the tidyverse package. All figures were created in R Studio using ggplot2 and VennDiagram packages. A paired *t*-test was performed in R to determine if there was a significant difference in the amount of protein extracted (i.e., total protein yield), the number of proteins and peptides identified, and the average sequence coverage (%), where a *p*-value < 0.05 was considered statistically significant. For statistical analyses with multiple comparisons (i.e., the number of unique membrane proteins annotated using gene ontology (GO) terms for cellular components and relative abundance changes), a paired *t*-test was performed with Benjamini-Hochberg (BH) correction, where an adjusted *p*-value < 0.05 was considered statistically significant. Protein covariance (CV) values can be found in Supplementary Data (Figure S6).

To reproducibly identify ORF products from each pre-extraction method (i.e., lyophilizing or flash-freezing cell pellets), the identified proteins from database searches needed to be identified in at least 3 biological replicates. Molecular weight, isoelectric point (pI), and the Grand Average of Hydropathy (GRAVY) scores were calculated in R Studio using the sum of masses of amino acid residues in a sequence, the European Molecular Biology Open Software Suite (EMBOSS) pKa scale from the Peptides package, and the Kyte-Doolittle hydropathy values for all residues in a sequence, respectively [26,27]. The Database for Annotation, Visualization and Integrated Discovery (DAVID, version 2025-04) was used to retrieve GO terms for cellular components [28].

## 3. Results

### 3.1. Assessment of Protein Extraction Yield

**Figure 1:**
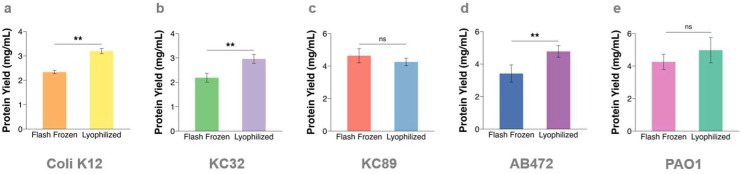
Total protein yield determined by paper-based assay using different homogenisation methods on *Escherichia coli* K12 (**a**), and *Klebsiella pneumoniae* isolates KC32 (**b**) and KC89 (**c**), *Acinetobacter baumannii* AB472 (**d**) and *Pseudomonas aeruginosa* PAO1 (**e**). Statistically significant differences are indicated by ‘*’ where two symbols indicate *p* < 0.01 and ‘ns’ indicates no significance (paired *t*-test, *n* = 5 for all isolates with the exception of KC89, where *n* = 3).

To determine the efficiency of homogenisation, total protein extraction yield was quantified using a paper-based protein assay. Lyophilization prior to homogenisation significantly improved the protein yield from most bacterial isolates, with the exception of KC89 and PAO1, where there is no significant difference between methods (Figure 1). Following protein extraction using UTC7, the extracts were centrifuged to remove insoluble material and cellular debris. For extracts from lyophilized cell pellets of all isolates, including KC89 and PAO1, there was minimal insoluble material. Following centrifugation for flash frozen cell extracts of all isolates, a noticeably large pellet of insoluble material was observed.

### 3.2. Assessment of Total Protein and Peptide Identifications, and Common and Unique Proteins Identified by Each Homogenisation Method

#### 3.2.1. *E.coli* K12

**Figure 2:**
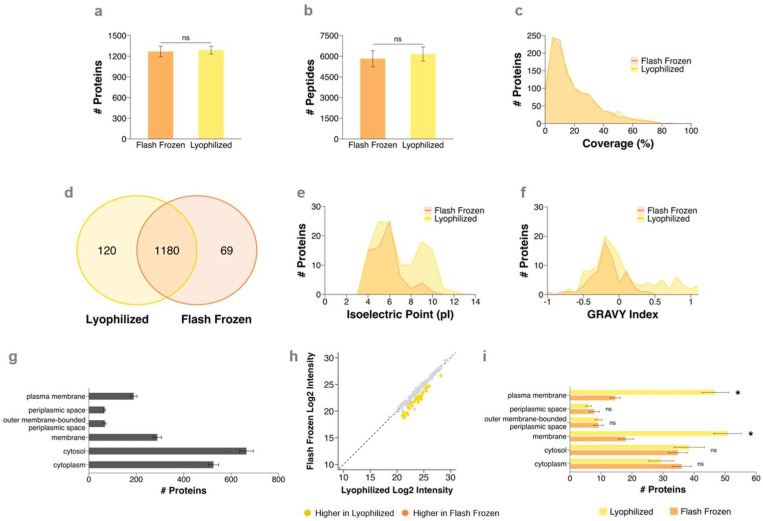
Number of protein and peptide identifications, protein sequence coverage distribution, common and unique proteins, physicochemical properties of proteins identified, and GO terms for cellular components among different homogenisation methods for Coli K12. Number of proteins (i.e., ORF products) (**a**) and peptides (**b**) identified in protein extracts resulting from different homogenisation methods where ‘ns’ indicates no significance (paired *t*-test, *n* = 5). Distribution of sequence coverage across common proteins identified (**c**). Venn diagram of identified protein groups in protein extracts resulting from different homogenisation methods, showing 1180 common proteins between methods, and 120 and 69 unique proteins identified in each method (**d**). Distribution of pI (**e**) and hydrophobicity (GRAVY index, (**f**)) of unique proteins identified. Cellular component GO terms for common (**g**) and unique proteins (**i**) identified in different homogenisation methods prior to extraction, statistically significant differences are indicated by ‘*’ where one symbol indicates *p* < 0.05 and ‘ns’ indicates no significance (paired *t*-test, BH correction, *n* = 5). Scatter plot of the log2-transformed intensities of common plasma membrane proteins identified in both homogenisation methods, where coloured circles indicate statistically significant changes in intensity (BH corrected *p* < 0.05, |log2 fold change| > 1), and grey circles indicate no significance. Dashed black line indicates a slope of m = 1, where any circles exactly on the dashed line indicate no changes in the log2 intensity between samples (**h**).

Despite the increased total protein extraction yield in the lyophilized cell extract, there was no significant difference in the total number of proteins (i.e., ORF products) or peptides identified by LC-MS/MS among the different extracts (Figure 2). In the flash frozen and lyophilized cell extracts, 1268 ± 76 and 1289 ± 57 proteins, and 5817 ± 583 and 6157 ± 510 peptides were identified for Coli K12, respectively (Figure 2a,b). Furthermore, lyophilizing cells prior to homogenisation did not improve overall proteome sequence coverage (Figure 2c). The average sequence coverage amongst common proteins identified in both the flash frozen and lyophilized cell extracts were 20.9% and 21.9%, respectively.

However, when comparing only the unique proteins identified by each homogenisation method, there seems to be an extraction bias towards a particular pI and hydrophobicity. For Coli K12, 120 and 69 proteins were uniquely identified in the lyophilized and flash frozen extracts, respectively (Figure 2d). Of these unique proteins, the lyophilized cell extract contained both acidic proteins (pH range of 4–6) and basic proteins (pH range of 8–10), in contrast to the flash frozen cell extract, which demonstrated a slight bias towards acidic proteins (Figure 2e). It has been previously demonstrated in *E. coli* that there is a strong link between the whole-proteome pI distribution and subcellular locations [29,30]. In addition to pI, hydrophobicity is also linked to subcellular location, and therefore, the hydrophobicity of the uniquely identified proteins was also determined by calculating the GRAVY index. The GRAVY index is a scale to measure hydrophobicity by averaging the hydropathy values of the amino acids in a protein sequence [27]. A positive GRAVY value indicates a more hydrophobic protein, whereas a negative GRAVY value indicates a more hydrophilic protein [27]. The lyophilized cell extract had a greater bias towards hydrophobic proteins (GRAVY index > 0) compared to the flash frozen cell extract, where mostly hydrophilic proteins (GRAVY index < 0) were uniquely detected (Figure 2f).

To confirm a potential relationship between physicochemical properties and subcellular locations, DAVID was used for functional annotation of the common and unique proteins identified (Figure 2g,i). The gene ontology (GO) term “cellular components” identified 47 additional unique plasma membrane proteins in the lyophilized group compared to the flash frozen group (Figure 2i). While a large number of AMR-related membrane proteins were detected in both methods, including outer membrane protein OmpC and TolC (accession no. P06996 and P02930, respectively) that are responsible for regulating the influx and efflux of molecules, lyophilizing prior to homogenisation resulted in a 16% increase in membrane proteins identified compared to flash-freezing prior to homogenisation (Figure 2g,i). Additionally, among the plasma membrane proteins detected in both the flash frozen and lyophilized samples, the majority showed no significant difference in abundance between methods; however, 33 proteins were significantly higher in lyophilized samples (BH corrected *p* < 0.05, |log2 FC| > 1), suggesting that lyophilization pre-homogenisation improved the extraction of numerous membrane proteins identified by each method (Figure 2h). To further confirm the presence of membrane proteins, transmembrane domain predictions were utilized (Supplementary Figure S1). Of the unique plasma membrane proteins identified in the lyophilized sample, 72% of them contain a transmembrane helix (Supplementary Data File S1 and Figure S1).

Unique AMR-related membrane proteins identified in the lyophilized cell extract included multi-drug resistance efflux pump protein mdtE (P37636), ABC transporter protein ModF (P31060), and intermembrane phospholipid transport proteins MlaD and MlaE (P64604 and P64606). Additionally, proteins involved in lipopolysaccharide (LPS) synthesis and assembly were also detected in the lyophilized Coli K12 cell extract, including LPS transport system proteins LptE and LptG (P0ADC1 and P0ADC6, respectively), LPS core heptose kinase waaY (P27240), and lipid A-core ligase waaL (P27243). The majority of unique proteins identified in the flash frozen cell extract are cytosolic or cytoplasmic proteins involved in DNA replication and repair pathways, and flagellar synthesis pathways (Supplementary Data File S1).

#### 3.2.2. *K. pneumoniae*

**Figure 3:**
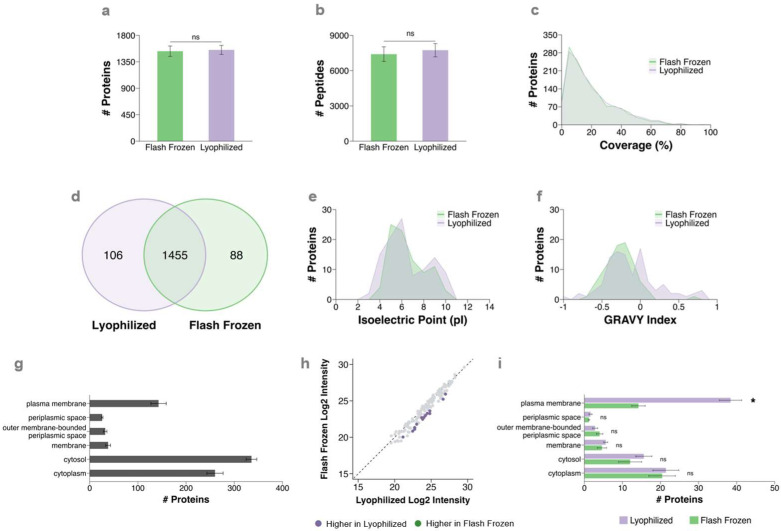
Number of protein and peptide identifications, protein sequence coverage distribution, common and unique proteins, physicochemical properties of proteins identified, and GO terms for cellular components among different homogenisation methods for KC32. Number of proteins (i.e., ORF products) (**a**) and peptides (**b**) identified in protein extracts resulting from different homogenisation methods where ‘ns’ indicates no significance (paired *t*-test, *n* = 5). Distribution of sequence coverage across common proteins identified (**c**). Venn diagram of identified protein groups in protein extracts resulting from different homogenisation methods, showing 1455 common proteins between methods, and 106 and 88 unique proteins identified in each method (**d**). Distribution of pI (**e**) and hydrophobicity (GRAVY index, (**f**)) of unique proteins identified. Cellular component GO terms for common (**g**) and unique proteins (**i**) identified in different homogenisation methods prior to extraction, statistically significant differences are indicated by ‘*’ where one symbol indicates *p* < 0.05 and ‘ns’ indicates no significance (paired *t*-test, BH correction, *n* = 5). Scatter plot of the log2-transformed intensities of common plasma membrane proteins identified in both homogenisation methods, where coloured circles indicate statistically significant changes in intensity (BH corrected *p* < 0.05, |log2 fold change| > 1), and grey circles indicate no significance. Dashed black line indicates a slope of m = 1, where any circles exactly on the dashed line indicate no changes in the log2 intensity between samples (**h**).

**Figure 4:**
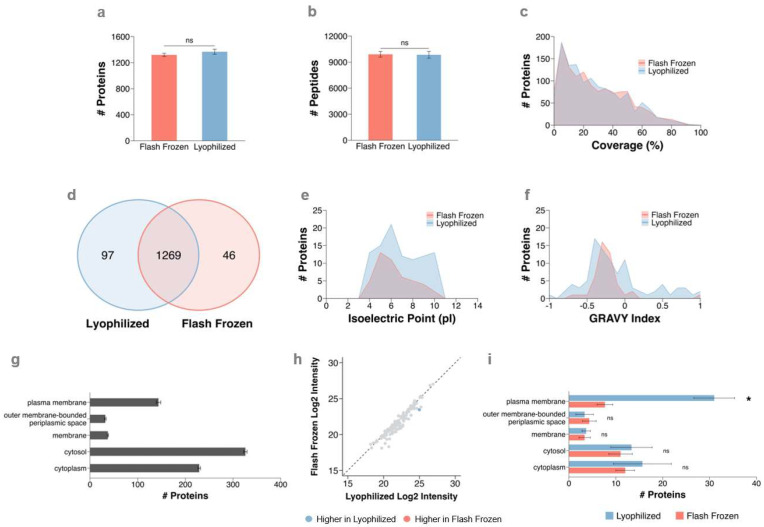
Number of protein and peptide identifications, protein sequence coverage distribution, common and unique proteins, physicochemical properties of proteins identified, and GO terms for cellular components among different homogenisation methods for KC89. Number of proteins (i.e., ORF products) (**a**) and peptides (**b**) identified in protein extracts resulting from different homogenisation methods where ‘ns’ indicates no significance (paired *t*-test, *n* = 3). Distribution of sequence coverage across common proteins identified (**c**). Venn diagram of identified protein groups in protein extracts resulting from different homogenisation methods, showing 1269 common proteins between methods, and 97 and 46 unique proteins identified in each method (**d**). Distribution of pI (**e**) and hydrophobicity (GRAVY index, (**f**)) of unique proteins identified. Cellular component GO terms for common (**g**) and unique proteins (**i**) identified in different homogenisation methods prior to extraction, statistically significant differences are indicated by ‘*’ where one symbol indicates *p* < 0.05 and ‘ns’ indicates no significance (paired *t*-test, BH correction, *n* = 3). Scatter plot of the log2-transformed intensities of common plasma membrane proteins identified in both homogenisation methods, where coloured circles indicate statistically significant changes in intensity (BH corrected *p* < 0.05, |log2 fold change| > 1), and grey circles indicate no significance. Dashed black line indicates a slope of m = 1, where any circles exactly on the dashed line indicate no changes in the log2 intensity between samples (**h**).

For both *K. pneumoniae* isolates, there was also no significant difference in the total number of proteins or peptides identified among the different extracts, or in overall proteome sequence coverage (Figure 3 and Figure 4). In the flash frozen and lyophilized cell extracts, 1532 ± 89 and 1553 ± 77 proteins, and 7403 ± 621 and 7739 ± 563 peptides were identified for KC32, respectively (Figure 3a,b). The average sequence coverage amongst common proteins identified in both the flash frozen and lyophilized cell extracts was 20.9 ± 1.1% and 21.9 ± 1.2%, respectively (Figure 3c).

There were 1455 proteins detected in both methods, and 106 and 88 proteins were uniquely identified in the lyophilized and flash frozen extracts, respectively (Figure 3d). Of the common plasma membrane proteins detected in each extract, the abundance of 15 proteins was significantly higher in lyophilized cell extracts compared to the flash frozen cell extracts (BH corrected *p* < 0.05, log2 FC > 1), further supporting an increase in the extraction efficiency of membrane proteins by lyophilizing cells pre-homogenisation (Figure 3g,h). When comparing the unique proteins identified by each homogenisation method in KC32, we also observed a small extraction bias towards hydrophobic proteins in the lyophilized cell extract, but no extraction bias towards acidic or basic proteins (Figure 3e,f). Of these unique proteins, 38 unique membrane proteins were identified when lyophilizing prior to homogenisation, resulting in a 15% increase in the detection of membrane proteins (Figure 3g,i). Of the unique plasma membrane proteins identified in the lyophilized sample, 50% of the proteins contain a transmembrane domain (Supplementary Data File S1 and Figure S2).

Unique AMR-related membrane proteins in the lyophilized cell extract include ferrous iron transport protein B (accession no. A0A0H3H420), multi-drug transporter component (A0A0H3GR16), outer membrane protein assembly factor BamE (A0A0H3GX19), and potassium efflux protein KefA and transport protein Kup (A0A0H3GSW4 and A0A0H3GPH2, respectively). Similar to Coli K12, the majority of unique proteins identified in the flash frozen cell extract are cytosolic or cytoplasmic DNA-binding proteins, including DNA methyltransferase proteins (A0A0H3GWR3 and A0A0H3GV12, respectively), and transcriptional regulator proteins EvgA, DsdC and PurR (A0A0H3GVU1, A0A0H3H4T0, and A0A0H3GUI1, respectively) (Supplementary Data File S1).

For KC89, in the flash frozen and lyophilized cell extracts, 1319 ± 24 and 1367 ± 38 proteins, and 9901 ± 323 and 9845 ± 390 peptides were identified, respectively (Figure 4a,b). The average sequence coverage amongst common proteins identified in both the flash frozen and lyophilized cell extracts were both 30.4% ± 0.6 or 0.8%, respectively (Figure 4c). Regarding the unique proteins identified in KC89, we also observed a slight extraction bias towards hydrophilic acidic proteins in the flash frozen cell extract, but no extraction bias towards proteins of a particular pI or hydrophobicity in the lyophilized cell extract (Figure 4e,f). In KC89, there were 97 and 46 proteins uniquely identified in the lyophilized and flash frozen cell extracts, respectively (Figure 4d). Of the proteins uniquely identified in the lyophilized cell extract, 31 proteins were characterized by the GO term ‘plasma membrane’, demonstrating a 15% increase in membrane protein detection (Figure 4g,i). Additionally, of the unique plasma membrane proteins identified in the lyophilized sample, 55% of the proteins contain a transmembrane domain (Supplementary Data File S1 and Figure S3).

Unique AMR-related membrane proteins identified in the lyophilized cell extract include integral membrane protein DedA (accession no. A0A0H3H138) and penicillin-binding protein 2 MrdA (accession no. A0A0H3GTX1). Both methods detected 140 plasma membrane proteins; however, only one protein showed a significantly higher log2 intensity in the lyophilized cell extracts compared to the flash frozen cell extracts (BH corrected *p* < 0.05, log2 FC > 1) (Figure 4h). As seen with Coli K12 and KC32, the majority of unique proteins identified in the flash frozen cell extract are cytosolic and cytoplasmic proteins, including those involved in DNA replication and transcriptional regulation, such as DNA polymerase III subunit delta and epsilon (accession no. A0A0H3GVI6 and A0A0H3GIQ4, respectively), DNA replication initiator protein DnaA (accession no. A0A0H3GRK5), and DNA-binding transcriptional regulator RstA (accession no. A0A0H3GP75) (Supplementary Data File S1).

#### 3.2.3. *Acinetobacter baumannii* AB472

**Figure 5:**
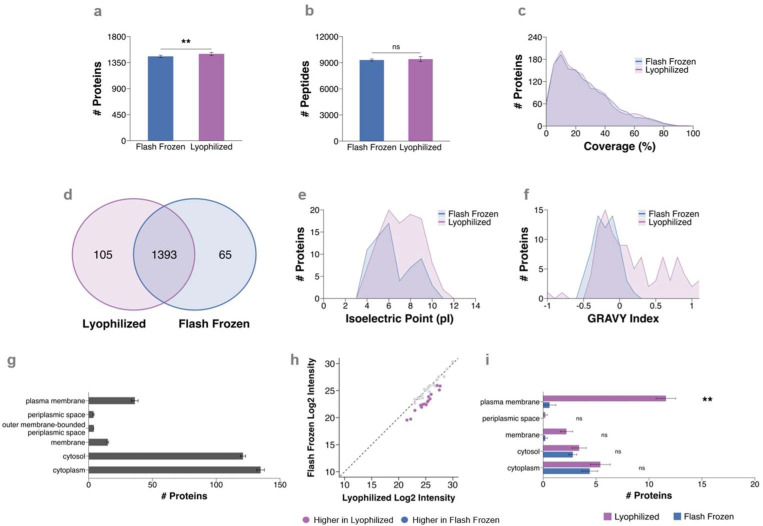
Number of protein and peptide identifications, protein sequence coverage distribution, common and unique proteins, physicochemical properties of proteins identified, and GO terms for cellular components among different homogenisation methods for AB472. Number of proteins (i.e., ORF products) (**a**) and peptides (**b**) identified in protein extracts resulting from different homogenisation methods, statistically significant differences are indicated by ‘*’ where two symbols indicate *p* < 0.01 and ‘ns’ indicates no significance (paired *t*-test, *n* = 5). Distribution of sequence coverage across common proteins identified (**c**). Venn diagram of identified protein groups in protein extracts resulting from different homogenisation methods, showing 1393 common proteins between methods, and 105 and 65 unique proteins identified in each method (**d**). Distribution of pI (**e**) and hydrophobicity (GRAVY index, (**f**)) of unique proteins identified. Cellular component GO terms for common (**g**) and unique genes (**i**) identified in different homogenisation methods prior to extraction, statistically significant differences are indicated by ‘*’ where two symbols indicate *p* < 0.01, respectively, and ‘ns’ indicates no significance (paired *t*-test, BH correction, *n* = 5). Scatter plot of the log2-transformed intensities of common plasma membrane proteins identified in both homogenisation methods, where coloured circles indicate statistically significant changes in intensity (BH corrected *p* < 0.05, |log2 fold change| > 1), and grey circles indicate no significance. Dashed black line indicates a slope of m = 1, where any circles exactly on the dashed line indicate no changes in the log2 intensity between samples (**h**).

In addition to the increased total protein extraction yield in the lyophilized cell extract, there was also a significant increase in the total number of proteins identified in the lyophilized extract. In the flash frozen and lyophilized cell extracts, 1468 ± 20 and 1501 ± 26 proteins were identified for AB472, respectively (Figure 5a). No significant difference was observed in the number of peptides detected (9314 ± 110 peptides for flash frozen and 9415 ± 285 peptides for lyophilized; Figure 5b), or the average sequence coverage for common proteins identified in each method (28.1 ± 0.5% for flash frozen and 27.9 ± 0.5% for lyophilized; Figure 5c).

Regarding the unique proteins identified in AB472, we also observed a slight extraction bias towards hydrophilic proteins in the flash frozen cell extract, but no extraction bias towards proteins of a particular pI or hydrophobicity in the lyophilized cell extract (Figure 5f,g). There were 105 and 65 proteins uniquely identified in the lyophilized and flash frozen cell extracts, respectively, and 1393 proteins identified in both methods (Figure 5d).

The *Acinetobacter baumannii* ‘reference’ proteome used for analyses in this study is the best available (i.e., most ‘complete’); however, annotation on the protein-level is poor. Only four out of the 3717 entries in the proteome have a subcellular component or biological process annotation. Therefore, for GO term annotation for cellular components via DAVID, gene names rather than accession numbers were used, as 479 of the 3717 entries have a subcellular component annotation. Among the plasma membrane ORF products detected in both the flash frozen and lyophilized samples, 17 ORF products were significantly higher in lyophilized samples (*p* < 0.05, |log2 FC| > 1), demonstrating improved extraction efficiency and detection (Figure 5h). Of the ORF products uniquely identified in the lyophilized cell extract, 11 proteins were characterized by the GO term ‘plasma membrane’, demonstrating a 32% increase in membrane protein detection (Figure 5g,i). Additionally, of the unique plasma membrane ORF products identified in the lyophilized sample, 57% of the proteins contain a transmembrane domain (Supplementary Data File S1 and Figure S4). Unique ORF products identified in the lyophilized cell extract include proteins involved in LPS synthesis and transport, such as lipid modification enzyme lpxO1 (accession no. A0ABX6CDF) and lptF and lptG proteins that make up the ABC transporter complex lptBFG (accession no. A0ABX6CCX1 and A0ABX6CB06, respectively) (Supplementary Data File S1).

#### 3.2.4. *Pseudomonas aeruginosa* PAO1

**Figure 6:**
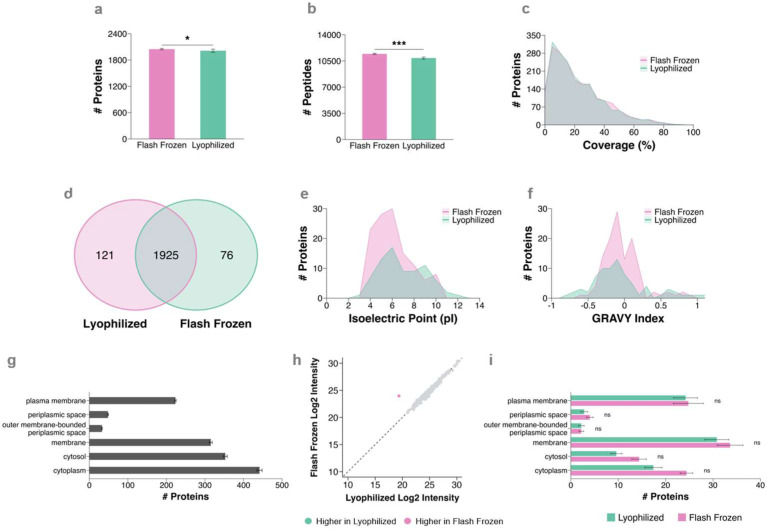
Number of protein and peptide identifications, protein sequence coverage distribution, common and unique proteins, physicochemical properties of proteins identified, and GO terms for cellular components among different homogenisation methods for PAO1. Number of proteins (i.e., ORF products) (**a**) and peptides (**b**) identified in protein extracts resulting from different homogenisation methods, statistically significant differences are indicated by ‘*’ where one and three symbols indicate *p* < 0.05 and *p* < 0.001, respectively, and ‘ns’ indicates no significance (paired *t*-test, *n* = 5). Distribution of sequence coverage across common proteins identified (**c**). Venn diagram of identified protein groups in protein extracts resulting from different homogenisation methods, showing 1925 common proteins between methods, and 121 and 76 unique proteins identified in each method (**d**). Distribution of pI (**e**) and hydrophobicity (GRAVY index, (**f**)) of unique proteins identified. Cellular component GO terms for common (**g**) and unique genes (**i**) identified in different homogenisation methods prior to extraction where ‘ns’ indicates no significance (paired *t*-test, BH correction, *n* = 5). Scatter plot of the log2-transformed intensities of common plasma membrane proteins identified in both homogenisation methods, where coloured circles indicate statistically significant changes in intensity (BH corrected *p* < 0.05, |log2 fold change| > 1), and grey circles indicate no significance. Dashed black line indicates a slope of m = 1, where any circles exactly on the dashed line indicate no changes in the log2 intensity between samples (**h**).

Despite there being no significant difference in total protein extraction yield, there was a significant increase in the total number of proteins and peptides identified in the flash frozen extract. In the flash frozen and lyophilized cell extracts, 2049 ± 16 and 2012 ± 36 proteins, and 11,447 ± 85 and 10,895 ± 133 peptides were identified, respectively (Figure 6a,b). There was no significant difference in the average sequence coverage for common proteins identified in each method (25.2 ± 0.4% for flash frozen and 24.6 ± 0.3% for lyophilized; Figure 6c).

Regarding the unique proteins identified in PAO1 through the different homogenisation methods, there was no bias towards a particular pI or hydrophobicity (Figure 6e,f). There were 121 and 76 proteins uniquely identified in the lyophilized and flash frozen cell extracts, respectively (Figure 6d). There was no significant difference in the number of plasma membrane proteins detected, in the intensity of the common membrane proteins detected or proteins with transmembrane domains amongst either homogenisation methods (Figure 6g–i; Supplementary Figure S5). The increase in the number of proteins and peptides detected in the flash frozen sample largely corresponds to an increase in cytosolic proteins, rather than membrane proteins. These unique proteins in the flash frozen extract mostly include proteins involved in transcriptional regulation and metabolic processes (Supplementary Data File S1).

## 4. Discussion

To generate a meaningful extraction comparison and ensure differences were due to homogenisation protocols, rather than biological variance of different cultures, isolates were grown up to late exponential/early stationary phase in LB, then divided into two aliquots. Two different pre-homogenisation methods were evaluated: (1) lyophilizing the cell pellet, hence referred to as the ‘lyophilized’ cell extract, and (2) flash freezing the cells in liquid nitrogen, hence referred to as the ‘flash frozen’ cell extract. Both samples were then bead-beaten, and the resultant homogenate was solubilized with UTC7 extraction buffer.

Bead-beating is one of the most commonly used mechanical disruption techniques for cell lysis and has been demonstrated to increase protein yields and identifications in numerous bacterial protein extraction workflows compared to detergent-based lysis methods alone [9,11,31,32,33]. Previously, it has been demonstrated in the microalga *Chlorella vulgaris* that lyophilizing harvested cells prior to lysis using various mechanical disruption methods significantly improved the extraction of soluble proteins. Lyophilization allowed for the detection of additional proteins involved in various metabolic pathways that were undetectable in the extracts from flash frozen harvested cells [19]. The authors hypothesized that lyophilizing weakens the connectivity of cell walls and allows for the release of ‘difficult to extract’ proteins during homogenisation. In this study, we quantitatively compare the homogenisation and resultant protein extraction efficiency in harvested bacterial cells that have been lyophilized or flash frozen to identify further improvements in bead-beating extraction protocols.

Here, we demonstrate that lyophilization prior to homogenisation increases total protein extraction yield from most Gram-negative bacterial isolates tested in this study, with the exception of KC89 and PAO1, where there was no significant difference in extraction yield between homogenisation methods (Figure 1). This is likely due to variations in cellular morphology and molecular characteristics between different bacterial species and strains, such as diversity in cell wall and membrane composition, or the presence of polysaccharide capsules. Nonetheless, lyophilizing KC89 or PAO1 cells prior to homogenisation and extraction did not result in a ‘loss’ in extraction yields. However, with all isolates, a noticeably large pellet of insoluble material was observed in the flash frozen extracts. The formed pellet would suggest inefficient homogenisation and protein extraction for flash frozen extracts. Based on this, we hypothesize that lyophilizing likely improves protein extraction yields due to cells becoming brittle and fragile during the dehydration process, resulting in a fine, uniform powder that is not achieved with flash frozen cells. The reduced size of the cell fragments increases the available surface area and exposure of individual protein molecules to enhance solubilization by chaotropes and surfactants, therefore improving protein extraction.

However, despite the increase in protein extraction yield, the number of proteins (i.e., ORF products) and peptides identified, as well as overall proteome sequence coverage, was generally not significantly different between pre-homogenisation methods. Some statistically significant differences were observed in the number of proteins identified in AB472 and PAO1; however, these differences are practically negligible, as it accounts for a >3% increase in total protein identifications. Furthermore, sequence coverage is dependent on several factors, including peptide ionization efficiency, sample complexity and dynamic range, and trypsin digestion efficiency, and therefore homogenisation efficiency may be independent [34].

When assessing the physicochemical properties of the unique proteins identified by each homogenisation method, there was an extraction bias towards proteins with a particular physicochemical property. It has been previously demonstrated in *E. coli* that there is a strong link between the whole-proteome pI distribution and subcellular locations [29,30]. The pI of cytoplasmic proteins cluster at a pH range of 5 to 6, and the pI of membrane proteins, particularly integral membrane proteins, cluster at a pH range of 8 to 9.5. This is due to the fact that bacterial membranes are negatively charged, and therefore the positive charge of basic amino acids creates a strong electrostatic attraction [29]. In addition to pI, hydrophobicity is also linked to subcellular location. There was no strong bias towards proteins of a particular pI; however, the lyophilized cell extracts had a greater bias towards hydrophobic proteins (GRAVY index > 0) compared to the flash frozen cell extracts, where mostly hydrophilic proteins (GRAVY index < 0) were uniquely detected. PAO1 was an exception to this observation, where there was no bias towards a particular pI or hydrophobicity. Given that membrane proteins have a high portion of hydrophobic regions, the combination of proteins with a higher pI and GRAVY index value indicates the improved extraction efficiency and detection of membrane proteins in the lyophilized cell extract of most of the GNB assessed in this study. Using GO terms for cellular components, we demonstrate an ~15% increase in the identification of plasma membrane proteins in all bacterial isolates with the exception of PAO1.

Changes within the bacterial cell membrane play a central role in AMR-associated molecular mechanisms. ABC transporter proteins identified in the lyophilized cell extract of *E. coli* are responsible for regulating the influx and efflux of metal ions, amino acids, lipids, and monosaccharides [35]. ABC transporters have also been previously suggested to play a role in the efflux of xenobiotics in various GNB, including those utilized in this study [35,36]. Loss of ABC transporters has been demonstrated to decrease metabolic fitness in pathogenic GNB strains due to host-specific nutritional deficiencies [35]. Improving the capacity to extract these membrane proteins can aid in understanding the molecular mechanisms associated with multi-drug resistant GNB by detecting the associated changes in the proteome due to antimicrobial exposure. We also uniquely identified numerous proteins in the lyophilized cell extract of *E. coli* and *A. baumannii* involved in LPS assembly and synthesis. LPS is a major virulence factor in GNB, allowing them to evade the host immune system during an infection by providing a protective capsule against serum complement proteins [37,38]. Colistin resistance in *A. baumannii* is also driven by changes in LPS structure and synthesis [39]. Furthermore, in *E. coli* and *K. pneumoniae*, we uniquely identified multidrug transporters and efflux pumps. Changes in the permeability of the outer membrane due to outer membrane porin (OMP) loss or modification, or the presence of efflux pumps, are well-known resistance mechanisms [40].

Membrane proteins have been notoriously described as challenging to extract compared to cytosolic or cytoplasmic proteins due to their strong interactions with the cell membrane, low abundance, and hydrophobic nature [41]. Therefore, improving the capacity to extract and detect membrane proteins can further aid in understanding the resistance mechanisms and molecular mechanisms associated with multi-drug resistant GNB. Lyophilization prior to homogenisation seems to overcome these challenges by weakening the cellular membrane integrity, releasing such proteins and therefore increasing accessibility of the extraction buffer to solubilize membrane proteins [19]. Membrane protein extraction typically involves an enrichment step, such as fractionation with multiple wash steps, chloroform/methanol partitioning, or using solutions of high alkalinity, such as sodium carbonate [41,42,43]. Here, we were able to improve the extraction of membrane proteins without an additional enrichment step following extraction.

However, AMR mechanisms involve a complex interaction of intracellular biological networks in addition to membrane proteins [40]. Many classes of antibiotics inhibit or interfere with DNA replication or protein translation, and therefore, detecting DNA-binding proteins or proteins involved in transcription or translation regulation can also aid in understanding resistance mechanisms [40]. In the flash frozen extracts of *K. pneumoniae* and *P. aeruginosa*, we uniquely identify numerous cytosolic proteins in the flash frozen extract, including DNA methyltransferases and other transcriptional regulators. Previously, it has been demonstrated that DNA methylation can modulate bacterial pathogenicity and resistance or susceptibility by regulating biofilm formation, a mechanism in which an osmotic barrier is created to prevent the penetration of antibiotics and their contact with bacterial cells [44,45]. Furthermore, antibiotic exposure induces a global stress response, altering numerous fundamental cellular processes and physiology, including slowed growth rates and changes in energy metabolism [46,47,48]. These mechanisms, often mediated by cytoplasmic proteins, also contribute to the global resistance phenotype of multi-drug resistant GNB but are often overlooked due to bias towards known resistance mechanisms.

There have been numerous attempts in the proteomics field to determine optimal homogenisation and extraction protocols for different sample types. Here, we demonstrate that despite having the same sample and extraction solutions, differences in cell treatment prior to homogenisation (i.e., lyophilizing vs. freeze drying) result in protein extraction and identification biases. An ‘all-in-one’ universal bacterial protein extraction protocol is likely non-existent, and the appropriate protocol depends on the specific aims of the research project. Lyophilization of bacterial cells may be considered the optimal method for most GNB proteomic analyses, particularly in the context of studying AMR mechanisms mediated by membrane proteins. However, there are still numerous proteins that were only detected in the flash frozen cell extract, and there are numerous AMR mechanisms mediated by cytosolic proteins. Alternatively, simply flash-freezing bacterial cell harvests prior to homogenisation may seem attractive due to the shortened protocol, as lyophilizing typically takes 16–24 h. It should also be noted that lyophilizing purified proteins has been shown to significantly change conformational structures, as structure and function depend strongly on interactions with water molecules, and therefore dehydration (i.e., lyophilization) may lead to destabilization [49,50]. This effect has not been investigated with cellular proteins prior to homogenisation and extraction; however, if structural proteomics or the ‘interactome’ are of interest, one should consider the use of lyoprotectants to preserve native structures and/or protein-protein interactions that may be altered during the drying process [51].

## 5. Conclusions and Future Directions

In conclusion, we demonstrated that lyophilizing Gram-negative bacterial cell pellets prior to homogenisation and protein extraction significantly improves protein extraction yields and increases the detection of membrane proteins. While the shotgun proteomics workflow used in this study measures proteins (i.e., ORF products) to assess protein extraction efficiency, using a urea-based buffer for protein extraction allows this protocol to be compatible with proteoform-based analyses, such as integrated Top-Down Proteomics (iTDP) (i.e., two-dimensional electrophoresis coupled to LC-MS/MS) to analyse proteoforms involved in AMR mechanisms. Furthermore, the annotation of protein databases is often based on gene-level information rather than proteoform-level information. For example, the *K. pneumoniae* and *A. baumannii* reference proteomes are poorly annotated, making the downstream analyses involving features beyond the genome challenging. For *K. pneumoniae*, of the 5728 entries in the UniProt reference proteome database, only six entries are in the Swiss-Prot reviewed database, with the remaining entries being predicted (~50%) or inferred from homology (~49%). For *A. baumannii*, of the 3717 entries in the UniProt reference proteome database, there are zero entries in the Swiss-Prot reviewed database, with the remaining entries being predicted (~55%) or inferred from homology (~45%). Therefore, conclusions regarding functional annotation analyses for any bacterial proteomic analyses should be interpreted with caution and validated or integrated with other data. Nonetheless, proteoforms, rather than proteins, are the functional units responsible for AMR mechanisms, and therefore, proteoform-level analyses should be utilized. The extraction workflow presented in this study is compatible with downstream proteoform analyses, such as 2D-PAGE, and therefore can be utilized in such analyses to gain a comprehensive understanding of resistance mechanisms and identify novel therapeutics and diagnostic targets on the proteoform level.

## Data Availability

The mass spectrometry data have been deposited to the ProteomeXchange Consortium via the PRIDE [52] partner repository with the dataset identifier PXD075198.

## Supplementary Materials

The following supporting information can be downloaded at: https://www.mdpi.com/article/10.3390/proteomes14030035/s1. Figure S1: Domain prediction of unique proteins identified in Coli K12. Figure S2: Domain prediction of unique proteins identified in KC32. Figure S3: Domain prediction of unique proteins identified in KC89. Figure S4: Domain prediction of unique proteins identified in AB472. Figure S5: Domain prediction of unique proteins identified in PAO1. Figure S6: Protein Covariance values. Supplementary Data File S1: Summary table of unique proteins identified in each homogenisation method (i.e., lyophilized (Lyo) and flash frozen (FF)) with their GO term annotated biological function, cellular component, and domain in all bacterial isolates.
