# Supplementary material for: Lyophilization Prior to Homogenisation and Extraction Increases Membrane Protein Detection in Gram-Negative Bacterial Proteomic Analyses
Source: Proteomes. 2026 Jul 15;14(3):35. doi: 10.3390/proteomes14030035 (PMC13398305; doi:10.3390/proteomes14030035)
Supplement: Supplementary file 1 [file proteomes-14-00035-s001.zip › Proteomes4218143_SupplementaryFigures.pdf]

Supplementary Materials

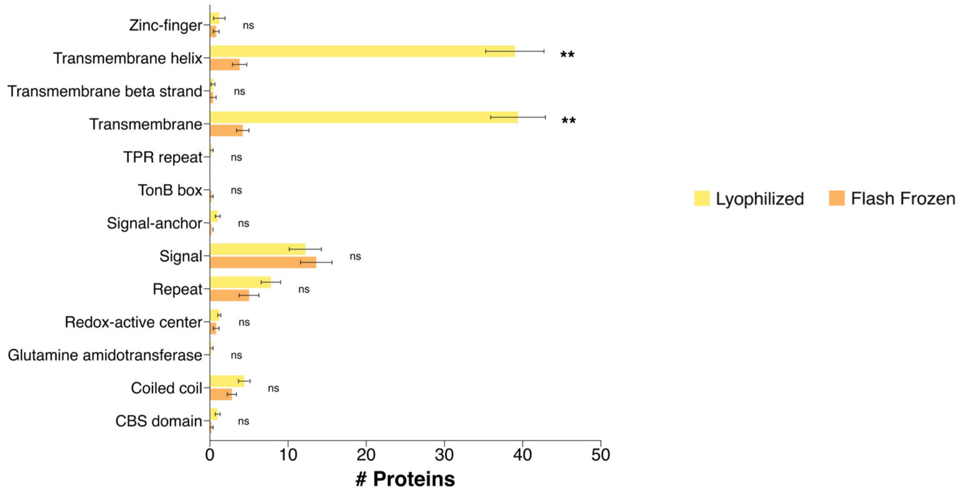

Figure S1: Domain prediction of unique proteins identified in ColiK12.

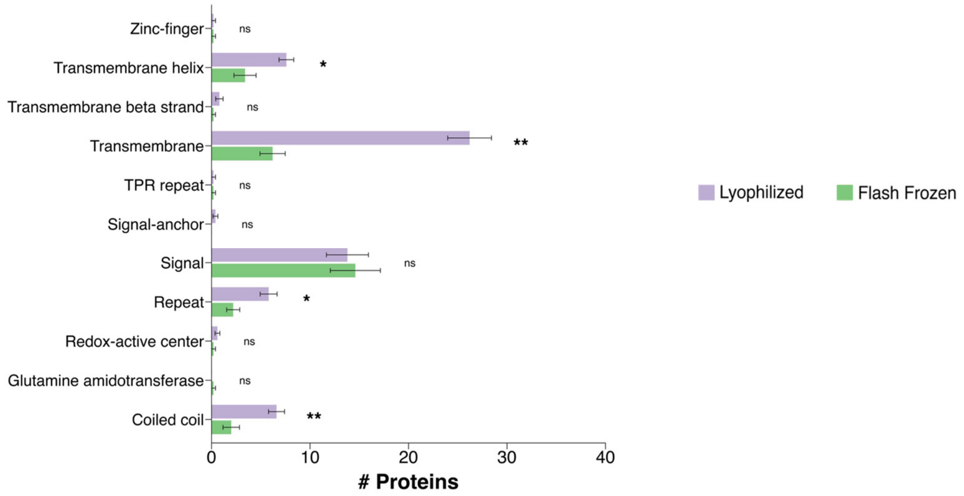

Figure S2: Domain prediction of unique proteins identified in KC32.

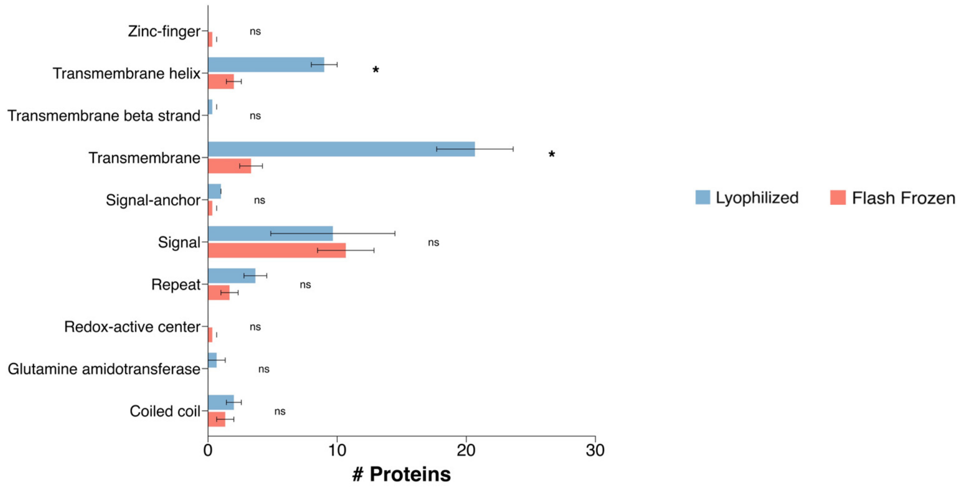

Figure S3: Domain prediction of unique proteins identified in KC89.

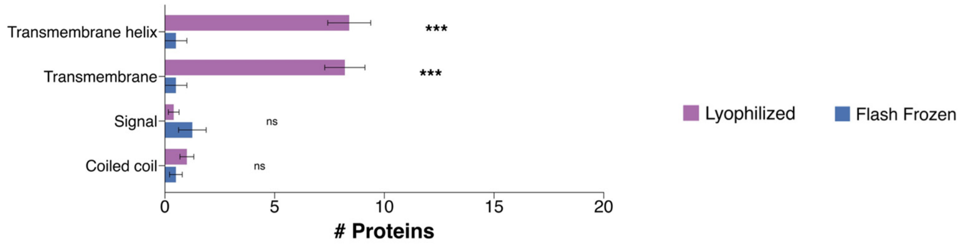

Figure S4: Domain prediction of unique proteins identified in AB472.

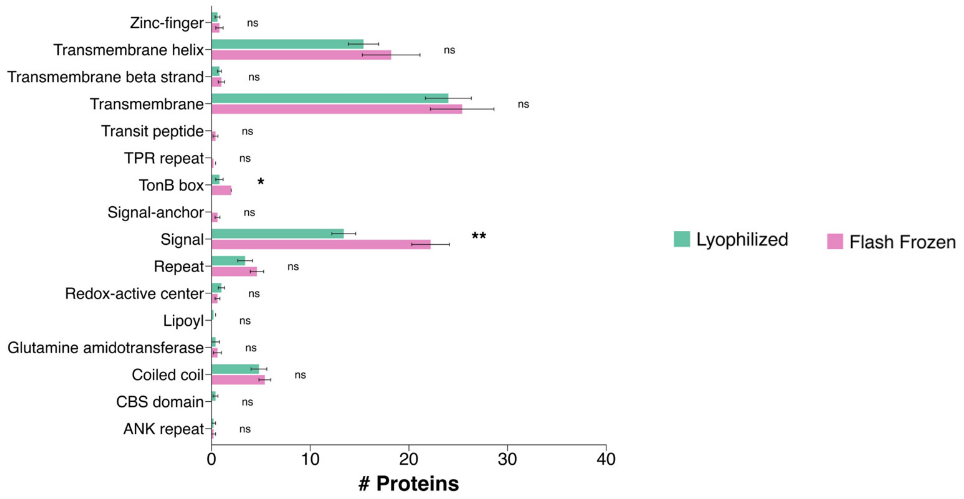

Figure S5: Domain prediction of unique proteins identified in PAO1.

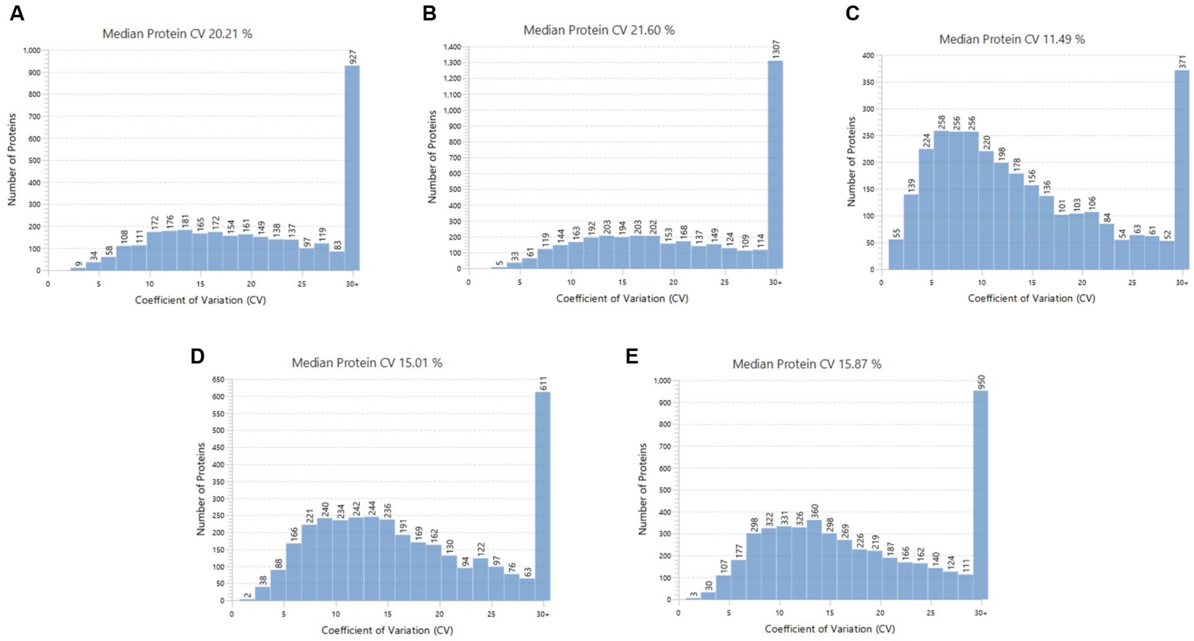

Figure S6: Protein covariance values. Protein CVs for Coli K12 (**A**), KC32 (**B**), KC89 (**C**), AB472 (**D**) and PAO1 (**E**) exported from PeaksStudio.
